# Routine 36‐week scan: diagnosis of fetal abnormalities

**DOI:** 10.1002/uog.29218

**Published:** 2025-03-25

**Authors:** A. Syngelaki, R. Mitsigiorgi, J. Goadsby, K. Hamed, R. Akolekar, K. H. Nicolaides

**Affiliations:** ^1^ Fetal Medicine Research Institute King's College Hospital London UK; ^2^ Department of Women and Children's Health, School of Life Course and Population Sciences King's College London London UK; ^3^ Fetal Medicine Unit Medway Maritime Hospital Gillingham UK; ^4^ Institute of Medical Sciences Canterbury Christ Church University Chatham UK

**Keywords:** fetal abnormality, prenatal diagnosis, pyramid of pregnancy care, third‐trimester screening, ultrasound examination

## Abstract

**Objectives:**

To investigate further the incidence and types of fetal abnormality identified at a routine 36‐week ultrasound examination, which had not been diagnosed in previous scans at 20 weeks and 12 weeks' gestation, and to report the fetal abnormalities that are diagnosed only postnatally.

**Methods:**

This was a prospective study of 104 151 women with a singleton pregnancy attending for a routine ultrasound examination at 35 + 0 to 36 + 6 weeks' gestation. In each case, a detailed examination was carried out for the diagnosis of fetal abnormality. All women had undergone a previous ultrasound examination at 19 + 0 to 23 + 6 weeks and 95 801 (92.0%) women also had a scan at 11 + 0 to 14 + 1 weeks. We excluded pregnancies with known aneuploidy. Fetal abnormalities were classified according to the affected major organ system, and the type and incidence of new abnormalities were determined.

**Results:**

There were four main findings of this study. First, in the study population, abnormality was identified in 2552 (2.5%) fetuses/neonates. Second, at the 36‐week scan, abnormality was detected in 2144 (2.1%) fetuses and the most common abnormalities first detected at the 36‐week scan were ventricular septal defect, unilateral or bilateral hydronephrosis, unilateral empty renal fossa (with or without pelvic kidney), unilateral or bilateral duplex kidney and mild ventriculomegaly. Third, 1341 (62.5%) of the fetuses with abnormality detected at the 36‐week scan had been diagnosed previously during the first or second trimester and therefore, the incidence of abnormality detected for the first time in the third trimester was 0.77% (803/104 151). The most common abnormalities that were diagnosed exclusively for the first time during the third‐trimester scan were ovarian cyst, achondroplasia, microcephaly, vein of Galen malformation and hematocolpos. Fourth, the incidence of abnormality detected for the first time postnatally was 0.39% (408/104 151). The most common abnormalities detected for the first time postnatally were polydactyly, oligodactyly or syndactyly, hypospadias/epispadias, mild talipes treated with physiotherapy, ventricular septal defect and isolated cleft palate. The most common abnormalities diagnosed exclusively for the first time postnatally were isolated cleft palate, anal atresia, atrial septal defect and esophageal atresia with fistula.

**Conclusion:**

A high proportion of fetal abnormalities are detected for the first time during a routine 36‐week scan. Such diagnosis and subsequent management, including the selection of timing and place for delivery and postnatal investigation, could potentially improve postnatal outcome. © 2025 The Author(s). *Ultrasound in Obstetrics & Gynecology* published by John Wiley & Sons Ltd on behalf of International Society of Ultrasound in Obstetrics and Gynecology.

## INTRODUCTION

The traditional approach to diagnosis of fetal abnormalities is to perform two routine ultrasound scans during pregnancy, the first at around 12 weeks' gestation and the second at around 20 weeks' gestation. There is now substantial evidence that a routine ultrasound scan should also be offered at 36 weeks' gestation. Such a scan in the late third trimester is useful for the prediction of small and large neonates^1^, ^2^, ^3^, ^4^, ^5^, diagnosis and management of non‐cephalic presentation^6^, ^7^, assessment of the risk for pre‐eclampsia and reduction of such risk by timed birth^8^, ^9^, ^10^, and prediction of adverse perinatal outcome^11^, ^12^, but also for diagnosis of previously undetected fetal abnormalities^13^, ^14^.

In a previous study^13^, we reported the findings from 52 400 singleton pregnancies that had undergone a routine ultrasound examination at 35 + 0 to 36 + 6 weeks' gestation. All pregnancies had a previous ultrasound scan at 19–23 weeks and 90% of pregnancies had also undergone a scan at 11–14 weeks^13^. The incidence of fetal abnormality was 1.9%, and 25% of these were detected for the first time at the 36‐week scan. The abnormalities detected exclusively for the first time at the 36‐week scan were ovarian cyst, microcephaly, achondroplasia, dacryocystocele and hematocolpos.

The objective of this study, which is considerably larger than our previous one^13^, was to investigate further the incidence and types of fetal abnormalities identified by a routine 36‐week ultrasound examination which had not been diagnosed previously in scans performed at 20 and 12 weeks' gestation, and to report the fetal abnormalities that were diagnosed only postnatally.

## METHODS

### Study population and design

This was a prospective study including 104 151 women with a singleton pregnancy who had undergone a routine ultrasound examination at 35 + 0 to 36 + 6 weeks' gestation at King's College Hospital, London, or Medway Maritime Hospital, Gillingham, UK, between March 2014 and November 2023. All women had previously undergone an ultrasound examination at 19 + 0 to 23 + 6 weeks. In addition, 95 801 (92.0%) women also had an ultrasound scan at 11 + 0 to 14 + 1 weeks. Gestational age was determined by the measurement of fetal crown–rump length at 11–14 weeks or fetal head circumference at 19–23 weeks^15^, ^16^. The inclusion criteria for this study were singleton pregnancy examined by ultrasound at 35 + 0 to 36 + 6 weeks' gestation and resulting in a live birth or stillbirth. We excluded pregnancies with aneuploidy diagnosed prenatally or postnatally. The current study population includes all 52 400 cases included in our previous study^13^. Data on pregnancy outcome were collected from the hospital delivery records and all prenatal and postnatal findings were recorded in a fetal database. This study constitutes an analysis of data derived from routine clinical examinations and therefore did not require ethics committee approval.

### Ultrasound examination

All ultrasound examinations were carried out according to standardized protocols by sonographers who had obtained the Fetal Medicine Foundation certificate of competence in ultrasound examination for fetal abnormalities or by trainees under the supervision of certified sonographers^17^, ^18^. The ultrasound examinations were performed primarily transabdominally using a 3–7.5‐MHz curvilinear transducer. However, in 2–3% of cases in which there were technical difficulties in obtaining adequate views transabdominally, which occurred at the 12‐week scan primarily, a transvaginal scan (3–9 MHz) was also carried out. The time allocated for the ultrasound examination of the fetus was 30 min.

#### First trimester

At 11–14 weeks, we aimed to obtain the following views: a transverse section of the fetal head to demonstrate the skull, midline echo and the choroid plexuses; a midsagittal view of the face to demonstrate the nasal bone, midbrain and brainstem; transverse views to demonstrate the orbits, upper lip and palate; a sagittal section of the spine to demonstrate the spine and overlying skin; a transverse section of the thorax with use of color Doppler to assess the four‐chamber view of the heart and outflow tracts and record blood flow across the tricuspid valve; and transverse and sagittal sections of the trunk and extremities to demonstrate the stomach, kidneys, bladder, abdominal insertion of the umbilical cord, all long bones, hands and feet.

#### Second trimester

During the second‐trimester scan, we aimed to obtain the following views: a transverse section of the head at the level of the cavum septi pellucidi and lateral ventricles; a suboccipitobregmatic view to examine the midbrain, cerebellum and vermis; a midsagittal view of the face to examine the nasal bone and exclude micrognathia; transverse views of the orbits, upper lip and palate; sagittal, coronal and transverse views of the spine; a sweep through the heart in the transverse plane to include four‐chamber view, outflow tracts and three‐vessel view (with and without color Doppler); transverse and sagittal sections of the thorax and abdomen to examine the lungs, diaphragm, liver, stomach, bowel, umbilical cord insertion, kidneys, bladder and ureters; and systematic examination of upper and lower limbs for length and shape of each bone, position and movement of each joint and examination of both hands and feet, including the digits. Examination of the genitalia was not a compulsory part of the protocol.

#### Third trimester

The third‐trimester scan was aimed primarily at assessing fetal growth, amniotic fluid volume and Doppler measurements in the uterine, umbilical and fetal middle cerebral arteries. The sonographers were also instructed to assess the fetal anatomy in the same systematic way as in the second trimester, but it was accepted that, depending on the fetal position, examination of the fetal face, sacrum and extremities may not be possible. In cases in which the fetal head was in an inappropriate position, it was manipulated using gentle external pressure to provide adequate assessment of the fetal brain anatomy.

In any trimester, all cases of suspected fetal abnormality were examined by a fetal medicine specialist and all cases of suspected fetal cardiac defect were examined by a fetal cardiologist. In such examinations, additional views to the standard abovementioned views were obtained. For example, in cases of suspected abnormality of the corpus callosum in the transverse view, the brain was also examined in the midsagittal plane.

### Outcome measures

We included all abnormalities diagnosed antenatally and in the neonatal period. All neonates were examined by a pediatrician before hospital discharge, but certain asymptomatic internal abnormalities could have been inevitably missed. We classified abnormalities according to the major organ system affected, as central nervous system, face, thorax, heart and great arteries, abdominal wall and gastrointestinal, urogenital, skeletal, multiple for those involving more than one organ system, tumor or other. Ventriculomegaly was classified according to atrial width, as mild (10–12.9 mm), moderate (13–14.9 mm) or severe (≥ 15 mm). A diagnosis of microcephaly was made if the fetal head circumference was 3 SDs below the normal mean for gestational age^16^. Hydronephrosis was considered to be present if there was pelvicalyceal dilatation with an anteroposterior diameter ≥ 10 mm. Polydactyly was considered to be present if the extra digit contained bone, and talipes was considered to be present if the fetus required postnatal treatment, with surgery, the Ponseti method or physiotherapy alone. We included all cases of abnormalities of the heart and great vessels but excluded cases of persistent left superior vena cava and aberrant right subclavian artery because these are variants of normal, rather than true defects. Cases with at least two different major heart defects were classified as complex.

## RESULTS

### Study population

A routine ultrasound examination at 35 + 0 to 36 + 6 weeks' gestation was carried out in 104 151 singleton pregnancies. At the time of the third‐trimester scan, median maternal age was 32.1 (interquartile range (IQR), 27.9–35.6) years, median weight was 79.0 (IQR, 70.7–90.0) kg and median body mass index was 29.0 (IQR, 26.1–32.9) kg/m^2^. White ethnicity was reported in 78 551 (75.4%) women, black in 14 757 (14.2%) women, South Asian in 5675 (5.4%) women, East Asian in 1980 (1.9%) women and mixed in 3188 (3.1%) women. Diabetes mellitus Type 1 or 2 affected 1107 (1.1%) women and conception was by *in‐vitro* fertilization for 3946 (3.8%) pregnancies.

### Fetal abnormalities

Abnormality was identified in 2552 (2.5%) fetuses/neonates within the study population. This included 1341 (52.5%) that had been diagnosed previously during the first and/or second trimester, 803 (31.5%) that were detected for the first time at the 36‐week scan and 408 (16.0%) that were detected for the first time postnatally (Table 1). The incidence of fetal abnormality detected for the first time at the 36‐week scan was 0.77% (803/104 151), while the incidence of postnatal diagnosis was 0.39% (408/104 151).

**Table 1:** Diagnosis of fetal abnormalities in 104 151 pregnancies undergoing routine ultrasound examination

		Stage at firstdiagnosis		
Defect	Total	T1 or T2	T3	PN
*Central nervous system*				
Microcephaly	9	0	9	0
Mild VM	127	31	96	0
Moderate VM	10	3	7	0
Severe VM	9	5	4	0
Complete or partial ACC	21	17	4	0
Septo‐optic dysplasia	4	4	0	0
Arachnoid cyst	34	6	28	0
Cerebellar hypoplasia	2	2	0	0
Vermian hypoplasia	15	14	1	0
Blake's pouch cyst	8	8	0	0
Schizencephaly, porencephaly or hydranencephaly	2	1	1	0
Venous sinus thrombosis	4	3	1	0
Vein of Galen malformation	3	0	3	0
Craniosynostosis	3	1	1	1
Encephalocele	1	1	0	0
Open spina bifida	15	15	0	0
Spina bifida occulta	3	2	0	1
Lipomyelomeningocele	1	0	0	1
*Face*				
Micropthalmia	4	1	0	3
Dacryocystocele	4	0	3	1
Cataract	2	0	0	2
CLP	56	54	0	2
Cleft lip only	33	23	0	10
Cleft palate only	25	0	0	25
Midfacial hypoplasia	2	1	0	1
Micrognathia	8	5	0	3
Unilateral aural atresia/dysplasia	4	0	0	4
*Thorax*				
CDH	17	13	2	2
Diaphragmatic eventration	1	0	0	1
CPAM	61	58	3	0
Pulmonary sequestration	6	6	0	0
Pleural effusion	5	2	3	0
*Heart and great arteries*				
AVSD	6	5	1	
VSD	331	237	62	32
Atrial septal defect	7	0	0	7
CoA or hypoplastic AA	29	19	8	2
Double AA	16	16	0	0
Right AA	59	59	0	0
AS	3	1	1	1
PS	11	6	2	3
Aortic atresia	1	1	0	0
Pulmonary atresia	3	3	0	0
HLHS	1	1	0	0
ToF	15	13	1	1
TGA	13	10	1	2
Ebstein's anomaly or tricuspid dysplasia	9	6	2	1
Complex heart defect	3	3	0	0
CCTGA	1	1	0	0
Left atrial isomerism	5	5	0	0
*Situs inversus* with dextrocardia	1	0	0	1
Uhl's syndrome	1	0	0	1
Ventricular diverticulum (left)	1	1	0	0
Arrhythmia	16	9	7	0
*Abdominal wall and gastrointestinal*				
Exomphalos	7	7		
Gastroschisis	38	38	0	0
Pentalogy of Cantrell	1	1	0	0
Esophageal atresia	7	5	2	0
Esophageal atresia with fistula	5	0	0	5
Esophageal duplication cyst	1	1	0	0
Duodenal atresia	2	1	1	0
Small bowel obstruction/atresia	2	1	1	0
Rectovaginal fistula	1	0	0	1
Intestinal malrotation/volvulus	1	0	0	1
Hirschsprung's disease	3	0	0	3
Anal atresia/imperforate anus	13	0	0	13
Abdominal cyst (other than ovarian)	32	19	11	2
Non‐visible gallbladder	3	2	1	0
Meconium peritonitis	2	2	0	0
Hepatosplenomegaly	1	0	1	0
Hepatic arteriovenous malformation	1	0	1	0
*Genitourinary*				
Hydronephrosis*	378	57	321	0
Hydronephrosis* with:				
Hydroureter	11	1	10	0
Contralateral ERF	3	3	0	0
Contralateral MCDK	6	5	1	0
Hydroureter without hydronephrosis	15	2	13	0
LUTO	4	3	1	0
Unilateral MCDK	63	56	7	0
Duplex kidney*	117	74	43	0
Duplex kidney* with:				
Hydroureter	4	1	3	0
Hydronephrosis*	9	6	3	0
Unilateral ERF (with or without pelvic kidney)	162	122	39	1
ERF with:				
Contralateral duplex kidney	1	0	1	0
Contralateral MCDK	1	1	0	0
Bilateral renal agenesis	3	3	0	0
Unilateral renal cyst	16	12	4	0
Horseshoe kidney	3	3	0	0
Bilateral polycystic kidney				
AD (adult type)	8	7	1	0
AR (infantile type)	3	1	2	0
Bladder exstrophy	2	1	0	1
Ovarian cyst	48	0	48	0
Hematocolpos	2	0	2	0
Hypospadias/epispadias	108	1	0	107
Ambiguous genitalia	9	3	0	6
*Skeleton*				
Hemivertebra/scoliosis	14	12	0	2
Diastematomyelia	1	1	0	0
Achondroplasia	7	0	7	0
Skeletal dysplasia				
Non‐lethal	4	3	1	0
Lethal	4	3	1	0
Arthrogryposis	2	2	0	0
Absent/hypoplastic limb	13	13	0	0
Ectrodactyly	2	2	0	0
Polydactyly, oligodactyly or syndactyly	141	34	0	107
Talipes treated with:				
Physiotherapy	67	28	2	37
Ponseti method	73	67	1	5
Surgery	32	30	2	0
*Tumor*				
Brain	1	0	1	0
Facial	3	1	2	0
Cardiac (rhabdomyoma)	8	1	7	0
Lymphangioma	7	5	2	0
Sacrococcygeal teratoma	2	1	1	0
Femur (fibrosarcoma)	1		1	0
*Other*				
Cervical cyst	1	1	0	0
Epidermolysis bullosa letalis	1	0	0	1
Hemophagocytic lymphohistiocytosis	1	0	0	1
*Multiple*				
ACC, CLP	2	2	0	0
ACC, cleft palate, talipes treated with Ponseti	1	0	0	1
ACC, polydactyly	1	1	0	0
Moderate VM, hydronephrosis*	3	0	3	0
Severe VM, ovarian cyst	1	0	1	0
Septo‐optic dysplasia, AS, PS, macroglossia	1	0	0	1
Blake's pouch cyst, hydronephrosis*	1	0	1	
Spina bifida, unilateral ERF	1	1	0	0
CLP, ambiguous genitalia	2	1	0	1
CLP, unilateral ERF	2	2	0	
Micrognathia, micropenis	1	0	0	1
Ear dysplasia, syndactyly	1	0	0	1
AS, unilateral duplex kidney	1	1	0	0
AS, micrognathia, arthrogryposis	1	0	1	0
CoA, CLP	1	1	0	0
Right AA, unilateral ERF	1	1	0	0
Right AA, unilateral duplex kidney	1	1	0	0
Ebstein's anomaly, unilateral ERF	1	1	0	0
ToF, hemivertrebra, talipes treated with Ponseti	1	1	0	0
ToF, right AA, unilateral ERF	1	1	0	0
AVSD, unilateral hypoplastic thumb	1	0	0	1
CPAM, unilateral ERF	1	1	0	0
Esophageal atresia, hydronephrosis* with hydroureter, hemivertebra	1	1	0	0
Duodenal atresia, unilateral ERF	1	1	0	0
Duodenal atresia, hemivertebra	2	2	0	0
Abdominal cyst (mesenteric), duplex kidney*	1	1	0	0
Abdominal cyst (adrenal), talipes treated with Ponseti	1	1	0	0
Imperforate anus, hypospadias, unilateral MCDK	1	0	0	1
Unilateral ERF, hemivertebra	2	2	0	0
Unilateral ERF, ovarian cyst	1	0	1	0
Unilateral duplex kidney, talipes treated with Ponseti	1	1	0	0
Hydronephrosis*, talipes treated with Ponseti	1	1	0	0
Hypospadias, talipes treated with physiotherapy	1	1	0	0
*Total*	2552	1341 (52.5)	803 (31.5)	408 (16.0)

Data are given as *n* or *n* (%).

* Cases were either unilateral or bilateral.

AA, aortic arch; ACC, agenesis of the corpus callosum; AD, autosomal dominant; AR, autosomal recessive; AS, aortic stenosis; AVSD, atrioventricular septal defect; CCTGA, congenitally corrected transposition of the great arteries; CDH, congenital diaphragmatic hernia; CLP, cleft lip and palate; CoA, coarctation of the aorta; CPAM, congenital pulmonary airway malformation; ERF, empty renal fossa; HLHS, hypoplastic left heart syndrome; LUTO, lower urinary tract obstruction; MCDK, multicystic dysplastic kidney; PN, postnatal; PS, pulmonary stenosis; TGA, transposition of the great arteries; T1, first trimester; T2, second trimester; T3, third trimester; ToF, tetralogy of Fallot; VM, ventriculomegaly; VSD, ventricular septal defect.

The most common abnormalities detected for the first time at the 36‐week scan were hydronephrosis, mild ventriculomegaly, ventricular septal defect, ovarian cyst, duplex kidney, unilateral empty renal fossa (with or without pelvic kidney) and arachnoid cyst. Defects that were detected exclusively for the first time at this scan included ovarian cyst, achondroplasia, microcephaly, vein of Galen malformation and hematocolpos.

The most common abnormalities detected for the first time postnatally were polydactyly, oligodactyly or syndactyly, hypospadias/epispadias, mild talipes treated with physiotherapy, ventricular septal defect and isolated cleft palate.

#### Central nervous system abnormalities

Many fetal central nervous system abnormalities identified at the 36‐week scan had already been diagnosed in the first or second trimester. However, most cases of mild or moderate ventriculomegaly and arachnoid cyst, as well as all cases of microcephaly and vein of Galen malformation, were first detected in the third trimester.

#### Facial abnormalities

Most of the fetal facial abnormalities observed at the 36‐week scan had already been identified during the first or second trimester. The most common facial abnormality was cleft lip and palate, with all cases initially diagnosed in the first or second trimester. In contrast, isolated cleft palate, cataract and ear abnormalities were only detected postnatally. There were four cases of dacryocystocele, three of which were diagnosed at the 36‐week scan and one which was diagnosed postnatally.

#### Thoracic abnormalities

Most cases of congenital diaphragmatic hernia (13/17) and congenital pulmonary airway malformation (58/61) seen at the 36‐week scan had been diagnosed previously. Two cases of congenital diaphragmatic hernia and one case of diaphragmatic eventration were diagnosed postnatally.

#### Heart and great artery abnormalities

Most abnormalities of the heart and great arteries observed at the 36‐week scan had already been diagnosed during the first and/or second trimester scan. The most common abnormalities were ventricular septal defect and right aortic arch. In 62/331 (18.7%) cases of ventricular septal defect the diagnosis was first made at the 36‐week scan, and in 32/331 (9.7%) cases it was first made postnatally. Similarly, in 8/29 (27.6%) cases of coarctation of the aorta or hypoplastic aortic arch the diagnosis was first made at the 36‐week scan, and in 2/29 (6.9%) cases it was first made postnatally. All cases of atrial septal defect and some cases of pulmonary or aortic stenosis, tricuspid valve defect, tetralogy of Fallot and transposition of the great arteries were diagnosed for the first time postnatally.

#### Abdominal wall and gastrointestinal abnormalities

The most common abdominal wall defect seen at the 36‐week scan was gastroschisis, for which all cases had been diagnosed previously at the first‐ or second‐trimester scan. The most common gastrointestinal abnormality seen at the 36‐week scan was abdominal cyst, for which around one‐third of cases were first diagnosed in the third trimester. Overall, there were 12 cases of esophageal atresia, of which seven were suspected in the second or third trimester due to a persistently small stomach; in five cases, there was atresia with a fistula, all of which were diagnosed postnatally because in the prenatal scans there was no polyhydramnios and the stomach appeared normal. One case each of duodenal atresia and small bowel atresia were not diagnosed at the routine 20‐week scan but rather in the late third trimester after presenting with polyhydramnios. All cases of imperforate anus, Hirschsprung's disease and intestinal malrotation/volvulus were diagnosed postnatally.

#### Genitourinary abnormalities

Genitourinary abnormalities represented the largest category of abnormalities in our population, comprising 38.2% (976/2552) of cases. They were also the abnormalities most frequently diagnosed for the first time at 36 weeks, accounting for 62.1% (499/803) of cases diagnosed in late gestation. The most common genitourinary abnormalities were hydronephrosis, unilateral empty renal fossa (with or without a pelvic kidney), and unilateral or bilateral duplex kidney; their detection rate at 36 weeks was 84.9% (321/378 cases), 24.1% (39/162 cases) and 36.8% (43/117 cases), respectively. Among genital abnormalities, ovarian cyst and hematocolpos were diagnosed exclusively in the third trimester. Prenatal examination of the genitalia was not a compulsory part of the protocol; 96.6% (113/117 cases) of hypospadias/epispadias and ambiguous genitalia were diagnosed only after birth.

#### Skeletal abnormalities

Most skeletal abnormalities seen at the 36‐week scan had already been diagnosed at the first‐ or second‐trimester scan. The most common were talipes and digital defects. However, all cases of achondroplasia were first detected in the third trimester. The majority of cases of talipes treated with physiotherapy alone and most cases of digital defect were diagnosed postnatally.

#### Tumors

The majority of fetal tumors were first seen at the 36‐week scan. The most common tumor was rhabdomyoma, of which 7/8 cases were first detected at the 36‐week scan.

#### Other abnormalities

There was one case each of cervical cyst, epidermolysis bullosa letalis and hemophagocytic lymphohistiocytosis. The former was diagnosed at the 20‐week scan and the other two were first diagnosed postnatally.

#### Multiple abnormalities

There were 40 fetuses with more than one abnormality and in 26 (65.0%) of these, the diagnoses were made during the 20‐week scan. In seven cases, the diagnoses were first made at the 36‐week scan and, in a further seven cases, the diagnoses were made postnatally.

## DISCUSSION

### Main findings

There are three main findings of this prospective study of 104 151 women with a singleton pregnancy undergoing routine ultrasound examination at 35 + 0 to 36 + 6 weeks' gestation. First, abnormality was identified in 2552 (2.5%) fetuses/neonates, including 408 detected only in the postnatal period. Second, the incidence of fetal abnormality detected for the first time in the third trimester was 0.77% (803/104 151). The most common abnormalities diagnosed exclusively for the first time during the third trimester were ovarian cyst, achondroplasia, microcephaly, vein of Galen malformation and hematocolpos. Third, the incidence of abnormality detected for the first time postnatally was 0.39% (408/104 151). The most common abnormalities diagnosed exclusively for the first time postnatally were isolated cleft palate, anal atresia, atrial septal defect and esophageal atresia with fistula.

### Comparison with findings from previous studies

#### Prevalence of new abnormalities

A previous screening study including 13 023 pregnancies undergoing a growth scan at 35 + 0 to 36 + 6 weeks' gestation reported a prevalence of 3.3 new abnormalities per 1000 cases, with the most common abnormalities being hydronephrosis, duplex kidney, mild ventriculomegaly and ovarian cyst^14^. This rate is lower than the 7.7 per 1000 cases found in our study, likely because our protocol included systematic screening for detection of fetal abnormalities. The same group undertook a meta‐analysis of 13 studies including 141 717 women undergoing a third‐trimester scan^19^. The 13 included studies comprised 10 in which the scan was carried out at about 32 weeks' gestation, one in which the scan was carried out at 24–40 weeks and two in which the scan was carried out at about 36 weeks. Only two of the studies undertook systematic examination of fetal anatomy, whereas, in the other 11 studies, the scan was carried out to assess fetal growth, and fetal abnormalities were incidental findings. The pooled prevalence of a new abnormality diagnosed at the third‐trimester scan was 3.68 per 1000 women, but the heterogeneity between studies was very high (*I* ^2^ = 88%).

#### Central nervous system abnormalities

The 76% detection rate of mild ventriculomegaly at the 36‐week scan in our study was similar to the 71% detection rate reported in a large cohort study of 40 192 pregnancies, in which the mean gestational age at diagnosis was 29.6 weeks^20^. Only 18% of our cases of arachnoid cyst were detected at the 20‐week scan; this is consistent with the results of a previous series in which only 25% of cases were identified at mid‐gestation^21^. For vein of Galen malformation, a recent review of 51 cases reported gestational age at diagnosis ranging from 22 to 37 weeks^22^; however, all of our cases were diagnosed at the 36‐week scan because the diagnosis necessitates the use of color Doppler^23^, which we routinely carry out in the third‐trimester scan, but not in the second‐trimester scan. For microcephaly, although there are previous studies reporting prenatal diagnosis in the second trimester^24^, ^25^, all nine cases in our study were first detected in the third trimester; this aligns with the accelerated brain development that occurs in the late second and third trimester, during which impaired brain growth becomes more noticeable as the head circumference fails to increase proportionately.

#### Heart abnormalities

The most common heart abnormality in our study was ventricular septal defect, of which 71% were diagnosed in the first or second trimester, 19% were diagnosed at the 36‐week scan and 10% were diagnosed postnatally. While the incidence of spontaneous intrauterine closure of second‐trimester ventricular septal defects ranges from 5% to 84%^26^, ^27^, a study showed that about half of isolated perimembranous ventricular septal defects were first detected at the third‐trimester scan^28^. Some cardiac lesions, such as major vessel stenosis and ventricular outflow tract obstruction, evolve with advancing gestational age^29^. In our study, 3/14 cases of aortic or pulmonary stenosis were first detected in the third trimester and 4/14 were diagnosed postnatally, while 8/29 cases of coarctation/hypoplastic aortic arch were first detected at the 36‐week scan and 2/29 were first detected postnatally. A previous study of 117 neonates with critical aortic stenosis reported that only 10 were diagnosed prenatally, likely because the obstruction developed after an initial normal second‐trimester scan^30^. A recent meta‐analysis reported that prenatal ultrasound diagnosis of coarctation of the aorta remains challenging due to subtle manifestations in the second trimester and the potential for physiological changes^31^.

#### Genitourinary abnormalities

Our data show that hydronephrosis was the most common genitourinary abnormality, with 84.9% diagnosed in the third trimester, consistent with the findings of previous studies^32^, ^33^. This is explained by the exponential increase in fetal urine production during the third trimester unmasking underlying urinary tract abnormality^34^. Similarly, 37% of our cases of duplex kidney and 87% of cases of isolated hydroureter were first detected at the 36‐week scan. Additionally, 39/162 cases of unilateral empty renal fossa (with or without pelvic kidney) were first detected in the third trimester, probably because, as noted in previous studies, the increased fetal size at this stage of pregnancy likely enhances the ability to detect such abnormalities^33^, ^34^.

As in our previous study^13^, all 48 cases of ovarian cyst were first diagnosed in the third trimester, consistent with a meta‐analysis of 299 cases that reported a median gestational age at diagnosis of 33 weeks^35^. This timing reflects the maturation of the fetal hypothalamic‐pituitary‐ovarian axis, which occurs after 29 weeks' gestation^36^, ^37^.

#### Gastrointestinal abnormalities

Esophageal atresia, duodenal atresia and bowel obstruction are identified indirectly via their manifestations of polyhydramnios and empty stomach, a ‘double‐bubble’ sign and distended proximal bowel loops, respectively. Polyhydramnios typically develops after 24 weeks, once the amount of swallowed amniotic fluid exceeds the absorptive capacity of the stomach and proximal duodenum, as fetal swallowing increases exponentially with gestation from about 10 mL at 20 weeks to 850 mL at term^38^. Previous studies reported a mean gestational age at diagnosis of 29 weeks for duodenal/jejunoileal atresia^39^, ^40^. None of our cases of imperforate anus were diagnosed prenatally because there are no direct sonographic signs, and examination of the ‘target sign’^41^ was not part of our protocol.

### Implications for clinical practice

Traditionally, the goals of a third‐trimester ultrasound examination have been to assess fetal growth and wellbeing, fetal presentation or placental position, with an aim to predict and potentially prevent adverse perinatal outcome. These objectives can be achieved more effectively when the scan is performed at 35–36 weeks rather than at 30–32 weeks' gestation. Large observational studies and randomized controlled trials comparing the findings of the two scans have demonstrated that identification of small and large neonates and prediction of pre‐eclampsia and adverse neonatal outcomes, including perinatal mortality and morbidity, are significantly increased at 36 weeks compared with 32 weeks' gestation^1^, ^4^, ^5^, ^42^, ^43^, ^44^. This is primarily because the majority of these outcomes occur at term; the predictive accuracy is highest when the scan is carried out closer to the time of delivery. Although fetal anatomy can be examined equally at 32 weeks, in healthcare settings with capacity for only one routine third‐trimester scan this should be performed preferably at 36 weeks rather than at 32 weeks. The incidence of fetal abnormalities diagnosed for the first time at 36 weeks' gestation was 0.77%, indicating that one new abnormality was detected for every 130 routine scans performed. Such a finding provides further support for a routine 36‐week anomaly scan, in addition to those performed at 12 weeks and 20 weeks' gestation.

In the case of abnormalities that are associated with severe impairment, such as severe ventriculomegaly and microcephaly, in countries in which late termination is legal, the parents may be offered this option. In cases of heart abnormality requiring immediate neonatal support, such as coarctation of the aorta and pulmonary or aortic stenosis, delivery should be scheduled in a center with pediatric cardiac expertise. Similarly, fetuses with diaphragmatic hernia are best delivered in centers with good facilities for neonatal respiratory support and pediatric surgery. In other cases, such as those with hydronephrosis, ventriculomegaly or arachnoid or ovarian cyst, pediatricians should be alerted to the need for appropriate postnatal investigation and follow‐up. For example, failure to diagnose various urinary tract abnormalities and prophylactic use of antibiotics until further investigation could result in irreversible renal damage.

### Strengths and limitations

A strength of our study is the systematic examination of fetal anatomy in a large population of women undergoing a routine third‐trimester scan by appropriately trained sonographers in units with expertise in fetal medicine.

A limitation of this study, and most previous studies, relates to the postnatal ascertainment of congenital abnormalities. Although the incidence of congenital abnormality diagnosed in the postnatal period has increased in this population compared with that in our previous studies^13^, ^17^, ^18^, reflecting greater efficiency in reporting neonatal outcomes, the identification of abnormalities in the postnatal period remains a challenge. Despite all neonates being examined by a pediatrician, certain internal abnormalities, particularly those that are asymptomatic, such as ventricular septal defects and unilateral empty renal fossa, can be missed. Another potential limitation relates to the general applicability of our results, as the routine ultrasound examinations were carried out within the framework of fetal medicine units with readily available expertise; consequently, in a routine ultrasound department, some of the abnormalities we detected could have been missed.

### Conclusions

A high proportion of fetal abnormalities are detected for the first time during a routine 36‐week scan. Such diagnosis and subsequent management, including the selection of timing and place for delivery and postnatal investigation, could potentially improve postnatal outcome.

## Data Availability

Research data are not shared.
